# Downregulation of MMP1 in MDS-derived mesenchymal stromal cells reduces the capacity to restrict MDS cell proliferation

**DOI:** 10.1038/srep43849

**Published:** 2017-03-06

**Authors:** Sida Zhao, Youshan Zhao, Juan Guo, Chengming Fei, Qingqing Zheng, Xiao Li, Chunkang Chang

**Affiliations:** 1 Department of Hematology, Shanghai Jiao Tong University Affiliated Sixth People’s Hospital, Shanghai, China

## Abstract

The role of mesenchymal stromal cells (MSCs) in the pathogenesis of myelodysplastic syndromes (MDS) has been increasingly addressed, but has yet to be clearly elucidated. In this investigation, we found that MDS cells proliferated to a greater extent on MDS-derived MSCs compared to normal MSCs. Matrix metalloproteinase 1(MMP1), which was downregulated in MDS-MSCs, was identified as an inhibitory factor of MDS cell proliferation, given that treatment with an MMP1 inhibitor or knock-down of MMP1 in normal MSCs resulted in increased MDS cell proliferation. Further investigations indicated that MMP1 induced apoptosis of MDS cells by interacting with PAR1 and further activating the p38 MAPK pathway. Inhibition of either PAR1 or p38 MAPK can reverse the apoptosis-inducing effect of MMP1. Taken together, these data indicate that downregulation of MMP1 in MSCs of MDS patients may contribute to the reduced capacity of MSCs to restrict MDS cell proliferation, which may account for the malignant proliferation of MDS cells.

Myelodysplastic syndrome (MDS) is a heterogeneous group of clonal disorders derived from hematopoietic stem and progenitor cells(HSPC), and is characterized by ineffective bone marrow haematopoiesis, peripheral blood cytopaenias and a risk of progression to acute myeloid leukaemia^1^. The bone marrow in low-grade MDS is characterized by increased apoptosis, whereas high-grade patients are characterized by accumulation of blasts. The aetiology of MDS has been mainly ascribed to molecular alterations of CD34 + HSPC^2^,^3^. However, the bone marrow (BM) microenvironment may also contribute to the pathogenesis of MDS^4^,^5^.

Mesenchymal stromal cells (MSCs) are key components of the BM microenvironment and play a crucial role in supporting and regulating HSPC^6^,^7^. In addition to their supportive effects, stromal cells may also facilitate apoptosis of hematopoietic cells in some pathological circumstances^8^,^9^. Mhyre *et al*. demonstrated that co-culture with stromal cells enhances apoptosis susceptibility and upregulates various genes involved in apoptosis in MDS hematopoietic cells and leukaemia cell lines^8^. Distinct genetic abnormalities have been identified in a portion of MDS-derived MSCs^10^,^11^. In addition, several cytokines, adhesion molecules and transcription factors have also been reported to be altered in MSCs of MDS patients^12^,^13^,^14^. However, whether and how these abnormalities are associated with the pathogenesis of MDS have not been clearly elucidated.

Among the mediators released from MSCs, matrix metalloproteinases (MMPs) are important regulators of the tumour microenvironment^15^,^16^. MMPs can affect multiple signalling pathways that modulate the biology of cells, thus exhibiting tumour-promoting or -suppressing effects in different circumstances^17^,^18^,^19^,^20^. We performed mRNA expression profiling of the MMP family in MSCs, and found that only matrix metalloproteinase 1 (MMP1) was downregulated in MDS-derived MSCs compared with normal control MSCs (Supplementary Fig. S1). Thus, MMP1 was chosen for use in subsequent studies. MMP1 has been reported to target protease-activated receptor 1 (PAR1) on the tumour cell surface and promote invasion and metastasis in breast cancer^21^,^22^. By targeting PAR1, MMP1 activates intracellular G proteins and downstream signaling, such as Gα_12/13_-Rho, p38 MAPK and ERK, thus potentially altering the biological activity of tumour cells^23^,^24^,^25^,^26^.

In the present study, the role of MMP1 in the interaction of MSCs and MDS cells was evaluated. MMP1 secreted from MSCs inhibits the growth and induces apoptosis of SKM-1cells and primary CD34 + cells from MDS patients through interaction with PAR1, which further activates p38 MAPK and downstream genes. Thus, downregulation of MMP1 in MDS-derived MSCs is associated with increased MDS cell proliferation.

## Results

### MDS cells proliferate to a greater extent on MDS-MSCs compared with normal control MSCs

**Figure 1:**
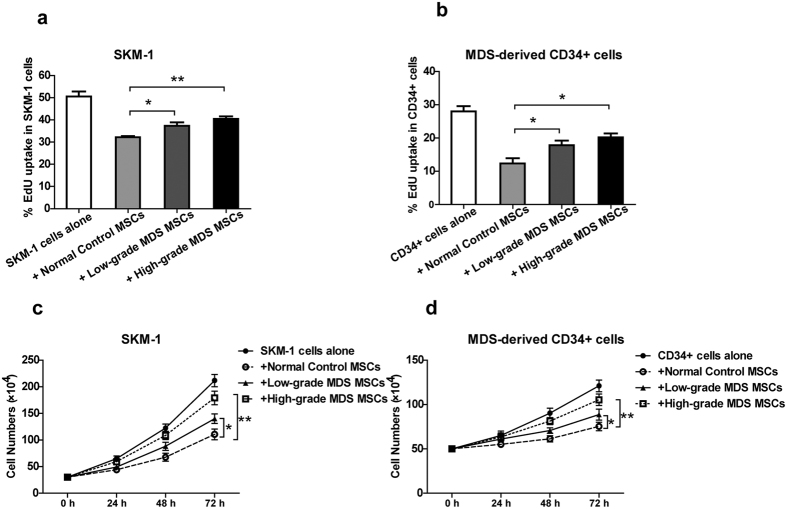
MDS cells proliferate to a greater extent on MDS-MSCs compared with normal control MSCs. SKM-1 cells (**a** and **c**) and MDS-derived CD34 + cells (**b** and **d**) were co-cultured with normal MSCs or MDS-MSCs or cultured alone. (**a** and **b**) The percentage of S phase cells was evaluated by the EdU assay after 72 h of culture. (**c** and **d**) Cells were counted with a haemocytometer at 24 h, 48 h and 72 h of culture. Normal MSCs and MDS-MSCs inhibited MDS cell proliferation. Both low-grade and high-grade MDS-MSCs exhibited reduced capacities to restrict the proliferation of MDS cells compared with normal MSCs. (Data represent the mean ± SEM from at least three independent experiments. *P < 0.05).

SKM-1 cells and MDS-derived CD34 + cells were cultivated alone or in the presence of normal MSCs or MDS-MSCs at a ratio of 5:2 and were tested for their proliferative activity after 72 h of culture by the EdU assay. In addition, cell numbers were counted using a haemocytometer at 24 h, 48 h and 72 h of culture. Co-culture with both normal MSCs and MDS-MSCs suppressed the proliferation activity of MDS cells compared with MDS cells cultured alone. Importantly, both the EdU assay and cell counting indicated that MDS cells proliferated to a greater extent on MDS-MSCs compared with normal control MSCs (Fig. 1).

### MMP1 as an inhibitory factor of MDS cell proliferation

**Figure 2:**
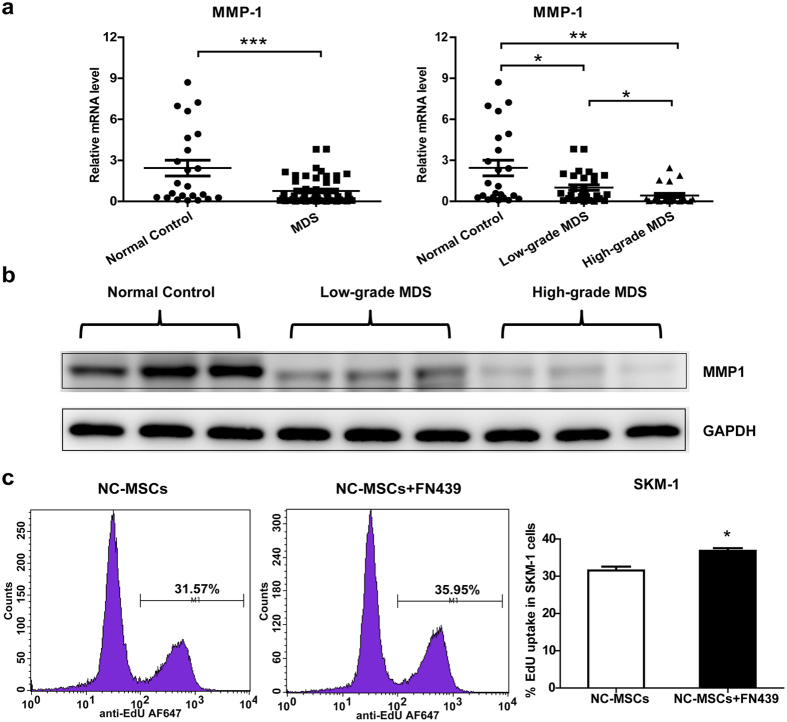
MMP1 is an inhibitory factor of MDS cell proliferation. (**a**) MMP1 mRNA expression in MDS-MSCs (n = 50; further classified as low-grade MDS (n = 29) and high-grade MDS (n = 21)) and normal control MSCs (n = 23) was measured by qPCR and compared with GAPDH. (**b**) MMP1 protein expression (54 kDa) in MDS-MSCs and normal control MSCs. (**c**)Addition of MMP1 inhibitor FN439 (5 μM) to normal control (NC) MSCs and SKM-1 co-culture increased the proportion of proliferating SKM-1 cells, as presented as flow cytometry plots (left) and a statistical figure (right). (Data represent the mean ± SEM from at least three independent experiments. *P < 0.05; **P < 0.01; ***P < 0.001).

MMPs secreted from stroma cells are important regulators of the tumour microenvironment. We performed mRNA expression profiling of MMP families (MMP1, MMP2, MMP3, MMP7, MMP8, MMP9, MMP11 and MMP12) in MSCs, and found that MMP1 was decreased in MDS-derived MSCs compared with normal MSCs (Supplementary Fig. S1 and Fig. 2a). In addition, high-grade MDS patients possessed lower levels of MMP1 than low-grade MDS patients. MMP1 mRNA expression was further confirmed through a comparison with another house-keeper gene (Supplementary Fig. S2a). The MMP1 protein levels were also decreased in MDS-derived MSCs, which is consistent with MMP1 mRNA expression (Fig. 2b). To test whether MMP1 is involved in the reduced capacity of MDS-MSCs to restrict the proliferation of MDS cells, we added the MMP1 inhibitor FN439 (5 μM) to normal MSCs and SKM-1 in co-culture. The addition of FN439 significantly increased the proportion of SKM-1 cells in the S phase (Fig. 2c). However, in the absence of MSCs, FN439 did not show any effects on MDS cell proliferation (Supplementary Fig. S2b). The above results suggest that MMP1 plays an important role in suppressing MDS cell proliferation in MSCs and MDS cells in co-culture.

### The inhibitory effect of MSCs on MDS cell proliferation is decreased when MMP1 is knocked down

**Figure 3:**
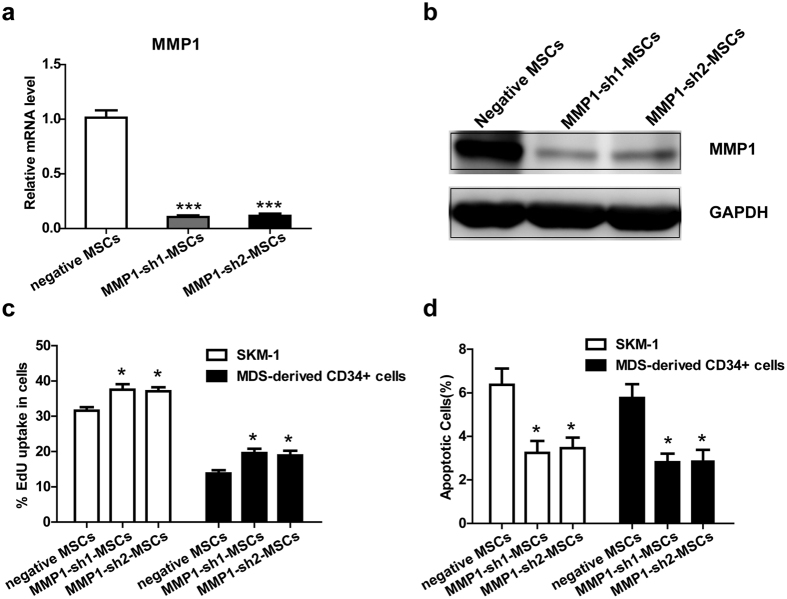
The inhibitory effect of MSCs on MDS cell proliferation is decreased when MMP1 is knocked down. The RNAi efficiency of MMP1 in MSCs was assayed by (**a**) qPCR and (**b**) western blotting before co-culture with MDS cells. MMP1 shRNA (both sh1 and sh2) decreased MMP1 expression of MSCs. (**c**) The percentage of MDS cells in S phase was evaluated by the EdU assay after co-culture with MMP1-KD MSCs (MMP1-sh1 MSCs and MMP1-sh2 MSCs) or negative MSCs (transfected with control lentiviruses) for 72 h. (**d**) The percentage of apoptotic MDS cells was assayed by Annexin V/PI dual staining after co-culture with MMP1-KD MSCs or negative MSCs for 72 h. (Data represent the mean ± SEM from at least three independent experiments. *P < 0.05; **P < 0.01; ***P < 0.001).

To further confirm that MMP1 is an important factor involved in the inhibitory effect of MSCs on cell proliferation, we constructed 2 retrovirus-based RNAi vectors that transfect MSCs with high efficiency. Normal MSCs were infected with the retroviral supernatant containing shRNA specific to human MMP1. On average, MMP1 was reduced by approximately 90%, as evaluated by real-time RT-PCR (Fig. 3a) and western blotting (Fig. 3b). We then evaluated the overall proliferation rate of MDS cells in the MMP1-knockdown (KD) group and negative control group. Similar to the results obtained from the MMP1 inhibitor assay, the proportion of MDS cells in the S phase was increased in the MMP1-KD group compared with the negative control group (Fig. 3c). In addition, co-culture with MMP1-KD MSCs resulted in decreased numbers of apoptotic MDS cells compared with negative control MSCs (Fig. 3d). Also, the proliferative proportion of CD34 + cells from healthy donors was increased and the apoptotic proportion was slightly decreased in the MMP1-KD group compared with negative control group (Supplementary Fig. S3a and b). In summary, the growth inhibition and apoptosis induction effects of MSCs on MDS cells were reduced when MMP1 was knocked down.

### MMP1 affects MDS cell proliferation and apoptosis through interaction with PAR1

**Figure 4:**
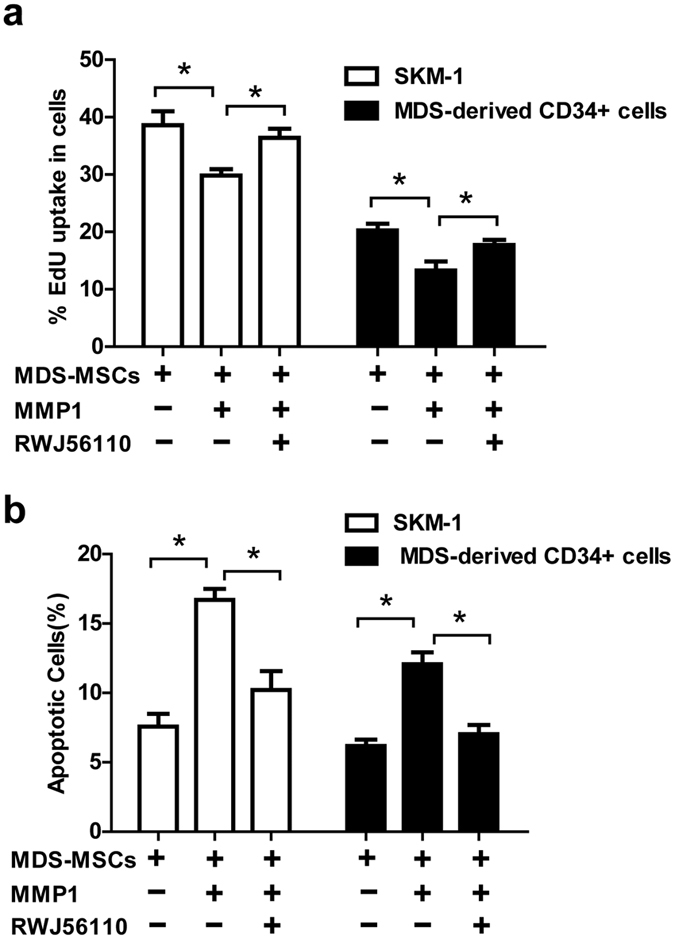
MMP1 affects proliferation and apoptosis of MDS cells through interaction with PAR1. In the MDS-MSC and MDS cells co-culture system, 1 nM exogenous activated MMP1 was added with or without pre-administration of RWJ56110 (5 μM). The percentage of proliferating MDS cells and apoptotic MDS cells was assessed by the EdU assay (**a**) and Annexin V/PI dual staining (**b**). (Data represent the mean ± SEM from at least three independent experiments. *P < 0.05).

PAR1 has been reported to be the target of MMP1. The proliferation of MDS cells was suppressed when exogenous activated MMP1 was added to MDS-MSCs and MDS cells in co-culture (Fig. 4a). Importantly, the proportion of apoptotic MDS cells, as measured by Annexin-V and PI staining, was significantly increased (Fig. 4b). To explore whether the growth suppressing and apoptosis inducing effects of MMP1 were mediated via PAR1, the PAR1 antagonist RWJ56110 was introduced prior to MMP1 addition. MMP1-induced growth inhibition and apoptosis was blocked by the PAR1 antagonist (Fig. 4a and b), thereby demonstrating that the effect of MMP1 on MDS cells was PAR1 dependent.

### MMP1/PAR1 exerts an apoptotic effect on MDS cells through the p38 MAPK pathway

**Figure 5:**
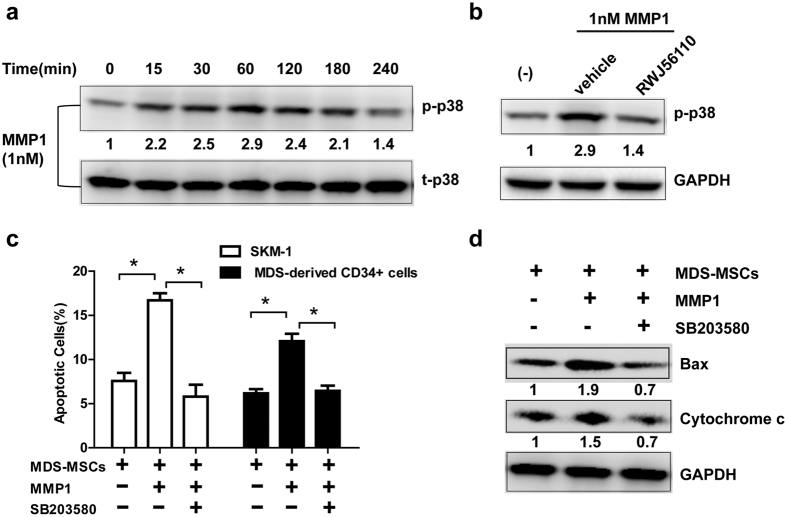
MMP1/PAR1 exerts apoptotic effect on MDS cells through the p38 MAPK pathway. (**a**) SKM-1 cells were starved overnight in serum free medium and stimulated with 1 nM MMP-1 over a period of 4 hours. Cell lysates were immunoblotted with anti-phospho-p38. Total p38 was used as the loading controls. Numbers below the band of p-p38 represented the relative expression levels of p-p38 at each time point compared with control according to gray scale analysis. (**b**) SKM-1 cells were pre-treated with 5 μM RWJ56110 and subsequently stimulated with 1 nM MMP-1. Cell lysates were immunoblotted with anti-phospho-p38. GAPDH was used as the loading control. Numbers below the band of p-p38 represented the relative expression levels of p-p38 of each group compared with control according to gray scale analysis. (**c**) In the MDS-MSCs and MDS cells co-culture system, 1 nM MMP1 was added with or without pre-treatment of p38 inhibitor SB203580 (10 μM). The percentage of apoptotic MDS cells was assessed by Annexin V/PI dual staining. (**d**) In the MDS-MSCs and SKM-1 cells co-culture system, 1 nM MMP1 was added with or without pre-treatment of the p38 inhibitor SB203580 (10 μM). Cell lysates of SKM-1 were immunoblotted with anti-Bax and anti-Cytochrome c. GAPDH was used as the loading control. Numbers below the band of Bax and cytochrome c represented the relative expression levels of Bax and cytochrome c of each group compared with control according to gray scale analysis. (Data represent the mean ± SEM from at least three independent experiments. *P < 0.05).

MAPKs have been established as downstream components of the MMP1-PAR1-G protein axis, and the phosphorylation of MAPKs in response to MMP1 has been shown to occur in platelets^23^. Therefore, we hypothesized that MMP1 can regulate apoptosis by activating the MAPK pathways upon interaction with PAR1. As predicted, treatment of SKM-1 cells with activated MMP1 caused a rapid and robust induction of p38 MAPK phosphorylation which peaked at 1 h upon stimulation and subsided by 4 h (Fig. 5a). RWJ-56110 inhibited the phosphorylation of p38 MAPK induced by MMP1 (Fig. 5b).

**Figure 6:**
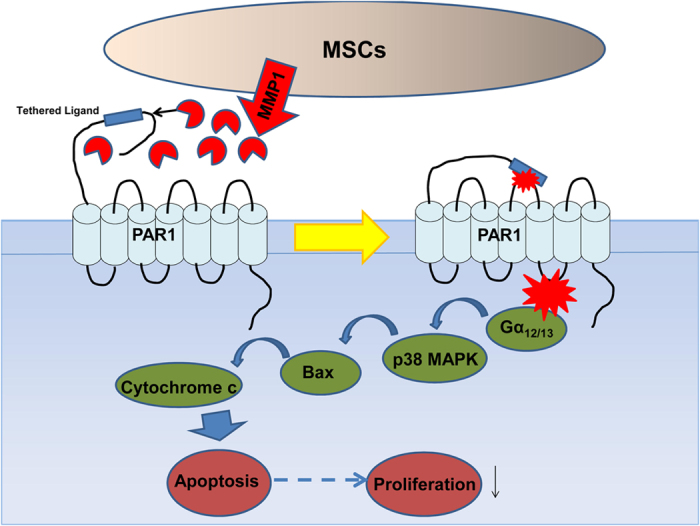
Model of MMP1/PAR1 interaction and subsequent activation of p38 MAPK signalling. MMP1 which is secreted by MSCs binds to and cleaves the extracellular N terminus of PAR1 to release a tethered ligand. Upon binding to the second extracellular loop, the ligand activates intracellular G proteins (Gα_12/13_) across the membrane and initiates activation of the p38 MAPK pathway, including the translocation of Bax and release of cytochrome c from the mitochondria, resulting in apoptosis and growth suppression. Therefore, downregulation of MMP1 in MDS-MSCs leads to reduced apoptosis resulting in increased MDS cell proliferation.

Next, we explored the significance of p38 MAPK signalling in the context of MMP1-induced apoptosis. We observed that the p38 inhibitor SB203580 completely reversed the proportion of apoptotic cells induced by MMP1 (Fig. 5c). In addition, the expression of pro-apoptotic proteins, such as Bax and cytochrome c which were increased in response to MMP1, were also blocked by SB203580 (Fig. 5d). These results strongly suggest that MMP1 confers cytotoxicity by activating the PAR1-p38 MAPK pathway. Thus, downregulation of MMP1 in MDS-derived MSCs leads to reduced apoptosis which may result in increased MDS cell proliferation (Fig. 6).

## Discussion

In this study, we demonstrated that MDS cells proliferated to a greater extent on MDS-MSCs compared with normal control MSCs. Downregulation of MMP1 of MDS-MSCs may partly account for this phenomenon. Either inhibition or knock-down of MMP1 in normal MSCs leads to increased MDS cell growth. MMP1 confers cytotoxicity by activating the PAR1-p38 MAPK pathway.

Recently, studies on MDS-derived MSCs mainly focused on their biological characteristics and hematopoietic support capacities. However, the interactions between MSCs and MDS cells are rarely reported. MSCs have been shown to suppress the proliferation of tumour cells by many researchers^27^. We demonstrated that MDS cells proliferated to a greater extent on MDS-MSCs compared with normal control MSCs, which may explain the possible pathogenesis of MDS.

Among the mediators released from MSCs, MMPs have been shown to be important regulators of the tumour microenvironment and various tumour-related processes, such as tumour growth, apoptosis, angiogenesis, invasion and metastasis^15^. MMP1 has been widely reported to be involved in tumour invasion; however, its regulation of cell apoptosis and proliferation has not been well covered in the literature. In this study, we demonstrated that MMP1 played an important role in apoptosis and that downregulation of MMP1 in MDS-MSCs may account for the reduced capacity to restrict proliferation and induce apoptosis of MDS cells. Consistently, Kittang *et al*.^28^ also observed decreased levels of MMP1 in high-grade MDS patients compared with low-grade MDS patients, which may support our findings given that high-grade MDS is characterized by the accumulation of blasts.

PAR1 is a G protein coupled receptor that is classically activated by thrombin^29^. Recently, MMP1 has been discovered to cleave and activate PAR1 at a non-canonical site, triggering Gα_12/13_-MAPK^24^. Our results demonstrate that a PAR1 antagonist is able to reverse the growth inhibition and apoptosis effects induced by MMP1, confirming the role of PAR1 in this process. Moreover, p38 MAPK was activated when MDS cells were treated with MMP1. Consistent with our data, Trivedi *et al*. also showed that exogenously added MMP-1 activated p38 MAPK and its substrate, MAPK-activated protein kinase-2 (MAPKAP-K2), in platelets^23^.

The role of p38 MAPK in apoptosis depends on the cell type and stimuli^30^. In some cell types, p38 MAPK has pro-apoptotic effects^31^,^32^. The possible mechanisms may involve the translocation or phosphorylation of Bcl-2 family proteins, resulting in the release of cytochrome c from the mitochondria^33^,^34^,^35^, caspase-8 activation induced by transforming growth factor-β^36^ and modulation of membrane blebbing and nuclear condensation^37^. In addition, growth arrest and DNA damage (GADD)-inducible genes also mediate the pro-apoptotic effects of p38 MAPK^38^. In this study, we found that inhibition of p38 MAPK reversed cell apoptosis induced by MMP1, indicating that the apoptosis effect induced by MMP1 was mediated by p38 MAPK. Furthermore, the Bax and cytochrome c protein levels, which were increased by MMP1, were also reversed by p38 MAPK inhibition, suggesting that the Bcl-2 family and cytochrome c may be involved in the mechanism of MMP1-PAR1-p38 MAPK-induced apoptosis.

In summary, our results demonstrate that MMP1 secreted from MSCs exhibits growth inhibition and apoptosis induction effects on SKM-1 cells and MDS-derived CD34 + cells by interacting with PAR1, which further activates p38 MAPK and downstream genes. Thus, reduced expression of MMP1 in MSCs from MDS patients had a decreased capacity to restrict the proliferation of MDS cells, which may account for the malignant proliferation of MDS cells.

## Materials and Methods

### Ethics Statement

The Ethics Committee of Shanghai Jiao Tong University Affiliated Sixth People’s Hospital approved all of the experimental protocols and methods described here. The study was performed according to the Declaration of Helsinki and the relevant ethical guidelines for research on humans. Informed consent was obtained from all subjects.

### Patients and control samples

Patients were diagnosed as MDS in accordance with the minimum diagnostic criteria established by the Conference on MDS^39^. A total of 50 patients with MDS were included in this study. Their characteristics are detailed in Table 1. Patients were classified for the study as “low-grade” (International Prognostic Scoring System (IPSS)-low/int-1) or “high-grade” (IPSS-int-2/high)^40^. A total of 23 healthy volunteers were used as controls and were matched by gender and age.

### Isolation and culture of BM-MSCs

Mononuclear cells (MNCs) were isolated from fresh BM aspirates and separated by a Ficoll-Paque Plus (GE Healthcare, Uppsala, Sweden). MNCs were seeded at an initial concentration of 1 * 10^6^ cells/mL and cultured in Human Mesenchymal Stem Cell Growth Medium (Cyagen Biosciences Inc., Guangzhou, China) supplemented with 10% foetal bovine serum (FBS), glutamine, and 100 U/mL Penicillin-Streptomycin at 37 °C with 5% CO_2_ in a fully humidified atmosphere. After 72 h, the culture medium was replaced and non-adherent cells were removed. Thereafter, medium was replaced every 3 to 4 d. Upon achieving greater than 80 to 90% confluency, cells were detached with 0.25% trypsin–EDTA (Gibco, Grand Island, NY, USA). At the third passage (P3), adherent BMMSCs were harvested and utilized for experimental analysis. BMMSCs were evaluated by cytometry for the absence of CD34 and CD45 antigens and the presence of CD73, CD90, CD105 and CD166.

### Isolation of CD34 + cells

CD34 + cells were isolated by Human CD34 Positive Selection Kit (StemCell Technologies, Vancouver, Canada) from BMMNCs according to manufacturer’s protocol. CD34+ cells purity was evaluated with Fluorescence Activated Cell Sorting (FACS) (BD Biosciences, Franklin Lakes, NJ, USA) and was >90%.

### Cell lines and culture

MDS cell line SKM-1 cells were gifted from Prof. Nakagawa. Cell lines were maintained in RPMI-1640 with 10% foetal bovine serum and penicillin(100 units/ml)/streptomycin(100 μg/ml). All cells were maintained in humidified air containing 5% CO_2_ at 37 °C.

### Reagents

Pro-MMP-1 and MMP inhibitor I (FN439) were obtained from Calbiochem (Darmstadt, Germany). Activation of pro-MMP-1 with APMA was performed as described previously^21^,^41^. The PAR1 antagonist RWJ-56110 was purchased from Tocris Bioscience (Bristol, UK). The p38 MAPK inhibitor SB203580 was purchased from Selleck Chemicals (Huston, USA).

### Proliferation assay

The cell proliferation rates were detected by EdU Flow Cytometry Assay Kits purchased from Life Technologies (Carlsbad, CA, USA). SKM-1 cells or primary CD34 + cells were treated with 10 μM EdU for 1 h and assessed according to the recommended staining protocol. Cells labelled with Alexa Fluor^®^ 647 azide were analysed on a flow cytometer using 633 nm excitation and a 660/20 nm bandpass emission filter.

Cells were counted using a haemocytometer. MDS cells from different time points were collected and resuspended in 1 ml of PBS. One part of 0.4% trypan blue and one part of cell suspension were mixed. A drop of the trypan blue/cell mixture was applied to a haemocytometer. The unstained (viable) cells were counted under a microscope in four 1 × 1-mm squares of one chamber, and the average number of cells per square was determined. The cell count was determined as follows: average cell count per square × dilution factor × 10 ^ 4 = cell count per ml.

### Apoptosis assay

The proportion of apoptotic cells was quantified by Alexa Fluor 488 Annexin V/propidium iodide (PI) dual staining (Invitrogen, Carlsbad, CA, USA). Cells were harvested, washed with phosphate-buffered saline (PBS), and re-suspended in 100 μL of binding buffer. Then cells were incubated with 5 μL of Annexin V and 1 μL of PI for 15 minutes in the dark at room temperature. The stained cells were analysed by flow cytometry as soon as possible.

### Real-time PCR

Total RNA was extracted using the RNeasy Mini Kit (QIAGEN, Hilden, Germany) following the manufacturer’s instructions. cDNA was synthesized using the Revert Aid TM First Strand cDNA Synthesis Kit (Fermentas, Burlington, Canada) according to the manufacturer’s protocol. PCR was performed with Real Master Mix (Takara, Dalian, China) on an ABI 7500 real-time PCR machine (Applied Biosystems, Foster, CA, USA). The primer sequences are listed in Table 2.

### Western blot analysis

Equal quantities of protein were analysed via 8 to 15% sodium dodecyl sulfate-polyacrylamide gel electrophoresis (SDS-PAGE) and transferred to poly (vinylidene difluoride) membranes. After incubation at 4 °C with primary antibodies against MMP1 (Proteintech Group, Rosemont, IL, USA), p-p38, p38, Bax, Cytochrome c and GAPDH (Cell Signalling Technologies, Boston, MA, USA) overnight, the blots were washed, exposed to corresponding HRP-conjugated secondary antibodies for 1 h, and finally detected by chemiluminescence reagents (Millipore, Billerica, MA, USA).

### MMP1 shRNA and cell transductions

Two pLenti X1 Puro-shDicer1-eGFP vectors and plenty X1 puro-shcontrol (negative vector) were constructed by Genechem Company (Shanghai, China). The target sequences against MMP1 were (5′-TTGTGGCTTATGGATTCAT-3′) and (5′-AAGATGAAAGGTGGACCAA-3′). The sequence inserted in the negative control was (5′- TTCTCCGAACGTGTCACGT-3′). The transfection was performed according to the manufacturer’s protocol. MSCs with different genes knocked down were named MMP1-KD MSCs and negative MSCs.

### Statistical analysis

All statistical analyses were performed using the SPSS 21.0 System (SPSS Inc., Chicago, IL, USA). Two independent samples were compared using Student’s t test. Multiple pairwise comparisons were performed using one-way analysis of variance (ANOVA). P < 0.05 was considered to be statistically significant.

## Additional Information

**How to cite this article**: Zhao, S. *et al*. Downregulation of MMP1 in MDS-derived mesenchymal stromal cells reduces the capacity to restrict MDS cell proliferation. *Sci. Rep.* **7**, 43849; doi: 10.1038/srep43849 (2017).

**Publisher's note:** Springer Nature remains neutral with regard to jurisdictional claims in published maps and institutional affiliations.

## Supplementary Material

Supplementary Information

## Figures and Tables

**Table 1:** Clinical characteristics of MDS patients.

Parameter		
Sex (median value)	Male	30
	Female	20
Age (median value)		61 (21–85)
WHO classification	RA	2
	RARS	6
	RCMD	17
	RAEB-1	12
	RAEB-2	9
	MDS-U	2
	5q- MDS	2
IPSS	<=1	29
	>1	21

**Table 2:** Primer sequences for quantitative real-time PCR.

Genes	Primer sequences(5′ to 3′)
MMP1	Forward primer AAAATTACACGCCAGATTTGCC
	Reverse primer GGTGTGACATTACTCCAGAGTTG
MMP2	Forward primer CCCACTGCGGTTTTCTCGAAT
	Reverse primer CAAAGGGGTATCCATCGCCAT
MMP3	Forward primer CTGGACTCCGACACTCTGGA
	Reverse primer CAGGAAAGGTTCTGAAGTGACC
MMP7	Forward primer GAGTGAGCTACAGTGGGAACA
	Reverse primer CTATGACGCGGGAGTTTAACAT
MMP8	Forward primer TGCTCTTACTCCATGTGCAGA
	Reverse primer TCCAGGTAGTCCTGAACAGTTT
MMP9	Forward primer TGTACCGCTATGGTTACACTCG
	Reverse primer GGCAGGGACAGTTGCTTCT
MMP11	Forward primer CCGCAACCGACAGAAGAGG
	Reverse primer ATCGCTCCATACCTTTAGGGC
MMP12	Forward primer GATCCAAAGGCCGTAATGTTCC
	Reverse primer TGAATGCCACGTATGTCATCAG
GAPDH	Forward primer GCACCGTCAAGGCTGAGAAC
	Reverse primer GTGGTGAAGACGCCAGTGGA
β-actin	Forward primer ATGTGGCCGAGGACTTGATTGC
	Reverse primer AGGATGGCAAGGGACTTCCTGTAA
