# Supplementary material for: Downregulation of MMP1 in MDS-derived mesenchymal stromal cells reduces the capacity to restrict MDS cell proliferation
Source: Sci Rep. 2017 Mar 6;7:43849. doi: 10.1038/srep43849 (PMC5338350; doi:10.1038/srep43849)
Supplement: Supplementary Information [file srep43849-s1.doc]

**Downregulation of MMP1 in** **MDS-derived mesenchymal stromal cells** **reduces the capacity to restrict MDS cell proliferation**

Sida Zhao, Youshan Zhao, Juan Guo, Chengming Fei, Qingqing Zheng, Xiao Li, Chunkang Chang*

Department of Hematology, Shanghai Jiao Tong University Affiliated Sixth People’s Hospital, Shanghai, China

Sida Zhao and Youshan Zhao contributed equally to this work.

Address correspondence to: Dr. Chunkang Chang E-mail: changchunkang@sjtu.edu.cn


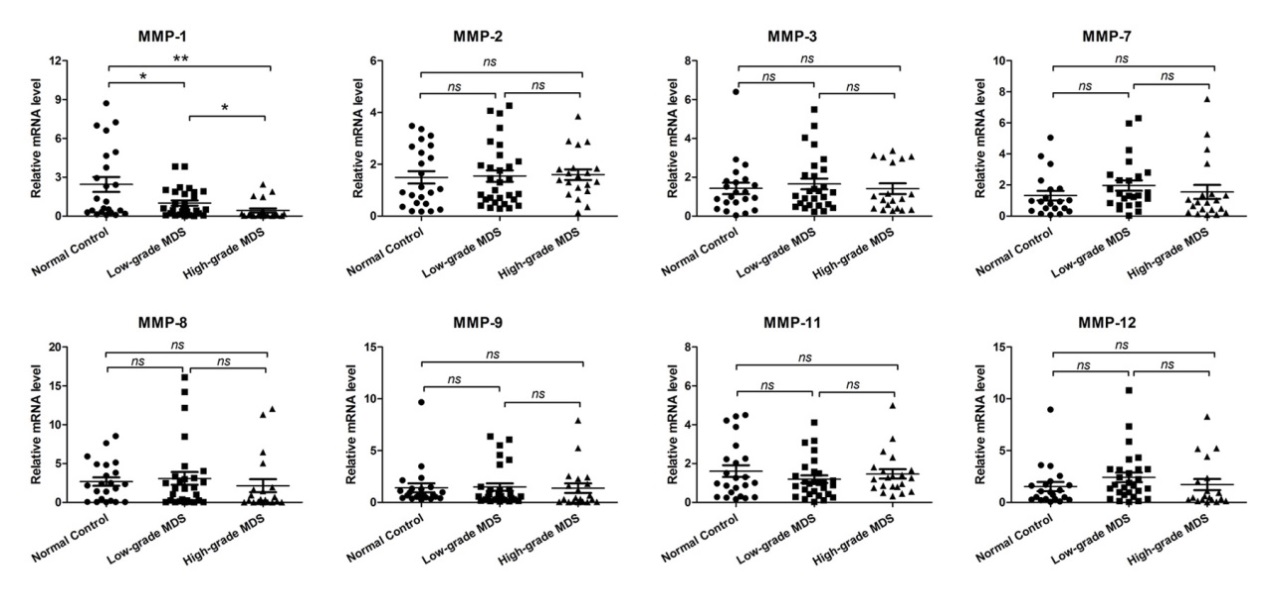
**Supplementary Figure. S1. The mRNA expression of the MMP family in MSCs.** MMP1, MMP2, MMP3, MMP7, MMP8, MMP9, MMP11 and MMP12 mRNA expression in MDS-MSCs (low-grade MDS (n=29) and high-grade MDS (n=21)) and normal control MSCs (n=23) was measured by qPCR and compared with GAPDH. There was no difference regarding MMP mRNA expression between MDS-MSCs and normal control MSCs except for MMP1. (Data represent the mean ± SEM from at least three independent experiments. *, P< 0.05; **, P< 0.01; ***, P< 0.001)


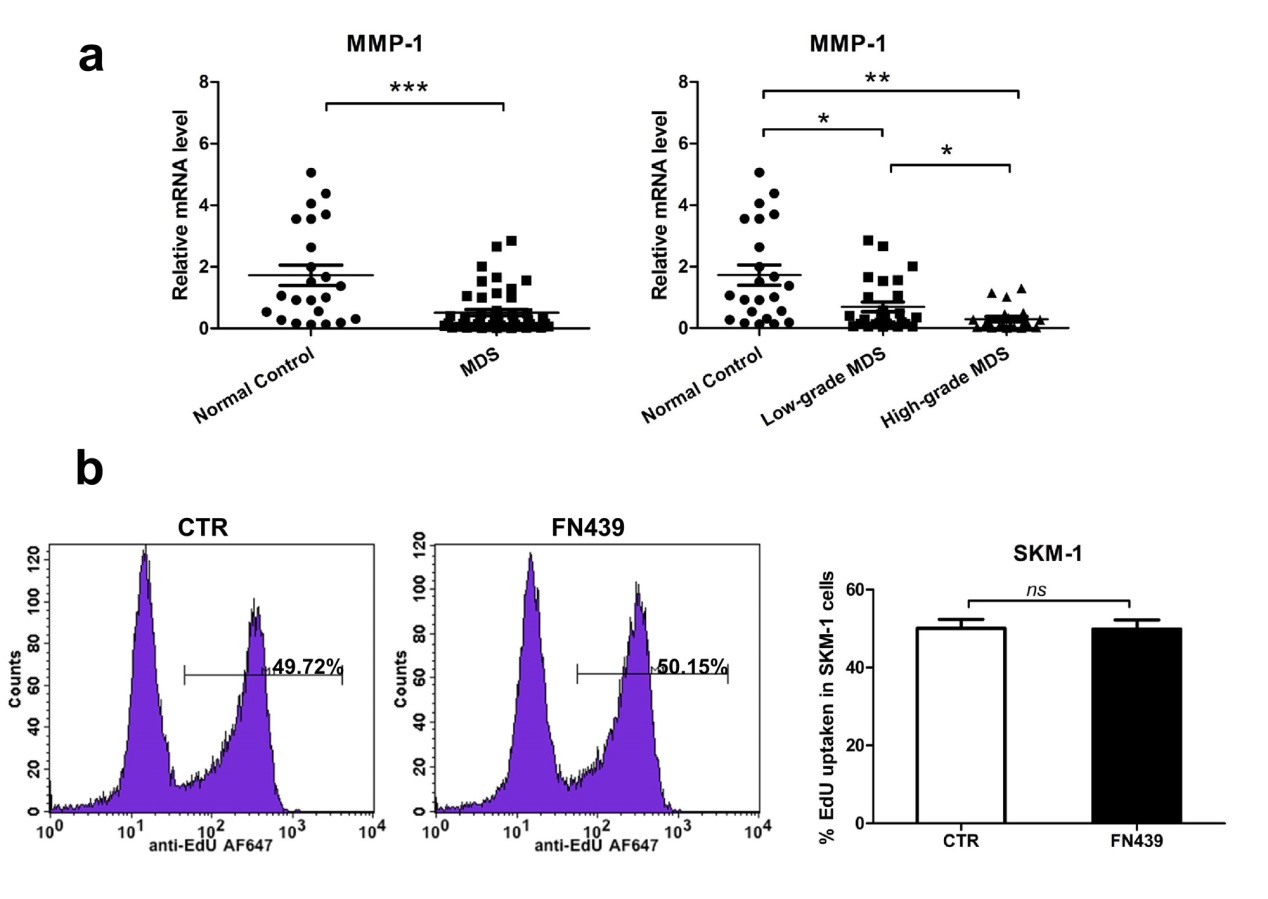


**Supplementary Figure. S2. MMP1 is an inhibitory factor of MDS cell proliferation.** (a) The mRNA expression of MMP1 in MDS-MSCs and normal control MSCs was further confirmed by comparison with β-actin. (b) MMP1 inhibitor FN439 (5 μM) did not exhibit any effect on the proliferation of SKM-1 cells in the absence of MSCs. The data are presented as flow cytometry plots (left) and a statistical figure (right). (Data represent the mean ± SEM from at least three independent experiments. *, P< 0.05; **, P< 0.01; ***, P< 0.001)


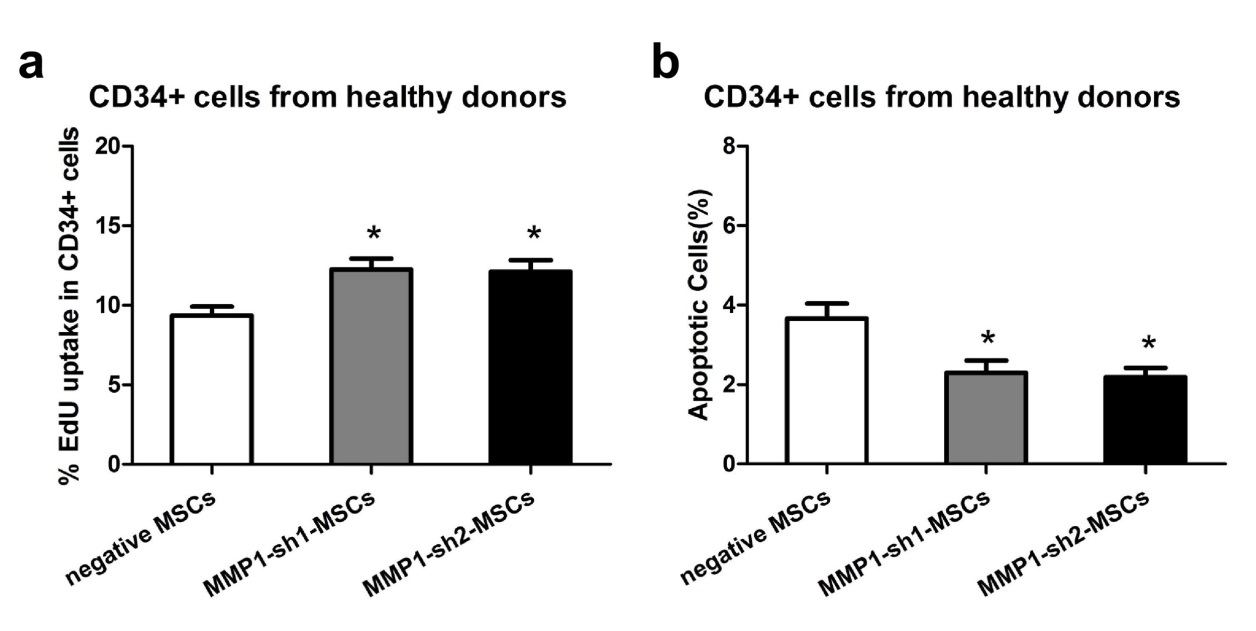


**Supplementary Figure. S3. The effect of MSCs on CD34+ cells from healthy donors when MMP1 is knocked down.** (a) The percentage of CD34+ cells from healthy donors in S phase was evaluated by the EdU assay after co-culture with MMP1-KD MSCs (MMP1-sh1 MSCs and MMP1-sh2 MSCs) or negative MSCs (transfected with control lentiviruses) for 72 h. (b) The percentage of apoptotic CD34+ cells from healthy donors was assayed by Annexin V/ PI dual staining after co-culture with MMP1-KD MSCs or negative MSCs for 72 h. (Data represent the mean ± SEM from at least three independent experiments. *, P< 0.05)
